# Climate and air pollution are associated with pediatric respiratory hospitalizations in São Paulo, Brazil

**DOI:** 10.3389/fped.2026.1899313

**Published:** 2026-09-23

**Authors:** William Cabral-Miranda, Dirceu Solé, Cauê Beloni, Celina de Almeida Lamas, Barbara Cristina Ferreira Ramos, Gustavo Falbo Wandalsen

**Affiliations:** Pensi Institute, Sao Paulo-SP, Brazil

**Keywords:** acute respiratory infections, air pollution, bronchiolitis, climate variability, ozone, pediatric hospitalization, seasonality, urban health

## Abstract

**Introduction:**

Environmental and climatic factors have been associated with pediatric respiratory diseases, but these associations may vary across specific clinical outcomes.

**Methods:**

We conducted a hospital–based study in the Metropolitan Area of São Paulo, Brazil, using pediatric emergency department visits and hospitalizations recorded in 2022. Residential addresses were geocoded and individual environmental exposures were estimated through inverse distance weighting (IDW) interpolation of air pollution and meteorological data. Multivariable logistic regression models were used to evaluate associations between environmental factors, seasonality, and two respiratory outcomes: bronchiolitis and unspecified acute respiratory infections.

**Results:**

Higher ozone levels, relative humidity, and spring seasonality were significantly associated with greater odds that a respiratory encounter corresponded to hospitalization for bronchiolitis (O_3_: OR = 1.012; 95% CI 1.005–1.019; relative humidity: OR = 1.016; 95% CI 1.005–1.027). For unspecified acute respiratory infections, lower temperatures were associated with greater odds that a respiratory encounter corresponded to hospitalization for this diagnosis (OR = 0.887; 95% CI 0.793–0.993), while greater odds were also observed during spring. Other environmental variables showed positive but non–significant associations.

**Discussion:**

Environmental and climatic factors showed differential associations with pediatric respiratory diseases. Disaggregating outcomes may improve the identification of disease–specific patterns of association and support targeted public health strategies in urban settings.

## Background

Urban living conditions especially for the most vulnerable populations, such as children, adolescents and the elderly have highlighted the impact of environmental factors on health. Air pollution has become an important public health concern for most cities worldwide, particularly in Latin America, where urban levels of air pollution are among the highest globally, making the potential health impact on children related to air pollution quite large (1).

The associations between air pollution and urban condition on health have been the focus of study in recent years. Exposure to pollutants such as airborne particulate matter (PM_10_- particulate matter with diameter equal or less than 10 *μ*m) and ozone (O_3_) has been associated with increases in hospital admissions due to respiratory conditions (2).

O_3_ is a potent oxidizer and highly toxic to the epithelial cells of the entire respiratory tract and acute exposure can induce transient physiological and biochemical changes while chronic exposure leads to cumulative damage or permanent decrease in airway function (3). Vinikoor-Imler et al. (4) concluded that there is evidence of increased risk for O_3_-related health associations among population subgroups with specific characteristics, including certain genotypes, pre-existing asthma, or reduced intake of key nutrients. High susceptibility was also observed among individuals at particular life stages (young and the elderly) as well as outdoor workers. Additionally, factors such as sex, socioeconomic status, and obesity were associated with evidence of increased vulnerability to O_3_-related health effects (4). A study conducted in Atlanta, Dallas, and St. Louis, United States of America, suggested that neighborhood-level socioeconomic status (SES) is a contributing factor to short-term vulnerability to O_3_-related pediatric respiratory morbidity. Nuanced relationships between neighborhood SES and O_3_-related respiratory health were observed in each city (3).

Air temperature is considered one of the main environmental factors associated with the burden of respiratory diseases, as daily variation and weather changes can affect the functioning of the human body and create conditions that increase the risk of hospital admissions (5).

A study conducted in China to identify the meteorological factors and model their associations on respiratory viruses across different regions of the country revealed that temperature, atmospheric pressure, vapour pressure, and rainfall had explanatory power for the majority of respiratory viruses in each region (6).

Dondi et al. (7) suggested that infant bronchiolitis may be one adverse effect of ambient air pollution on human health and that high levels of air pollutants could increase the risk of hospitalization, implying an increased risk of more severe cases.

Climatic profiles are important determinants of pediatric hospital admissions, as seasonal variations shape healthcare demand. Globally, increased health system burden during seasonal peaks of acute viral bronchiolitis is well documented (8).

The increased relative risk of hospitalization due to acute bronchiolitis has been associated with high temperatures and elevated vapor pressure, which is an indicator of absolute humidity. For PM_10_, the significant association observed at high concentrations appears to be immediate but not long-lasting (9). A prospective cohort study conducted from 2019 to 2020 in a pediatric emergency department in Milan, Italy, collected data on infants younger than 1 year of age with bronchiolitis. Daily exposure to PM₁₀ was positively associated with bronchiolitis severity score (10). Another study that assessed the risk of acute respiratory infections in children following exposure to air pollution, carried out a time-stratified case-crossover analysis to examine the association with high concentrations of air pollutants. An increased risk of unspecified acute upper respiratory infections was observed in relation to O₃, NO₂, and particulate matter with diameter equal or less than 2.5 μm (PM₂.₅). These findings suggest that exposure to air pollution may be associated with an increase the risk of respiratory illnesses in children within 24 h (11).

The Metropolitan Area of São Paulo provides a particularly relevant setting for investigating these associations because it is the largest metropolitan region in South America, with more than 21 million inhabitants, intense vehicular traffic, marked urban environmental heterogeneity, and persistent exposure to atmospheric pollutants. These characteristics create substantial spatial variability in air pollution and meteorological conditions, allowing the investigation of disease-specific environmental exposures in a large pediatric population. Moreover, although São Paulo shares environmental challenges with other large urban centers worldwide, evidence using individual residential exposure estimates and clinically confirmed pediatric outcomes from Latin American megacities remains scarce.

Despite growing evidence on the associations of air pollution and climatic factors on pediatric respiratory diseases, most studies have evaluated aggregated respiratory outcomes, potentially masking disease-specific patterns. Furthermore, evidence from Latin American megacities using individual residential geocoding and spatially estimated environmental exposures remains limited. Therefore, this study aimed to assess how environmental and seasonal factors are associated with distinct respiratory outcomes (unspecified acute respiratory infections and bronchiolitis) among children in the Metropolitan Area of São Paulo.

## Methods

### Study design and data sources

This is a hospital-based study conducted in the Metropolitan Area of São Paulo, Brazil, using data from emergency department visits and hospital admissions at Sabará Children's Hospital between January and December 2022. The study period comprised a complete calendar year, allowing the inclusion of all seasons and their corresponding climatic variability while ensuring consistency in clinical, environmental, and geocoded data collection. Because the objective was to investigate disease-specific environmental associations rather than interannual temporal trends, a one-year study period was considered appropriate for the proposed analyses. Sabará Children's Hospital was selected because it is a large tertiary pediatric referral hospital serving patients from multiple municipalities across the Metropolitan Area of São Paulo. The institution maintains a standardized electronic medical record system with complete clinical information and residential addresses, enabling individual-level geocoding and spatial linkage with environmental exposure estimates. Unlike our previous work, which evaluated aggregated respiratory diagnoses (ICD-10 Chapter J), the present study focused on unspecified acute respiratory infections (ICD-10 J06.9) and bronchiolitis (ICD-10 J21). Unspecified acute respiratory infections represented the most frequently recorded respiratory diagnosis in the hospital database during the study period, whereas bronchiolitis was selected because of its high clinical relevance as one of the leading causes of hospitalization among infants and young children. Analyzing these two outcomes separately allowed the investigation of whether environmental and climatic factors were differentially associated with common outpatient respiratory infections and a clinically severe lower respiratory disease. The clinical database included patient age, sex, date of attendance, hospitalization, and International Classification of Diseases (ICD-10) codes. Only cases with successfully geocoded residential addresses, meeting predefined accuracy thresholds, were retained for analysis. As a pediatric hospital, medical care is only provided to children under 18 years of age.

During the study period, Sabará Children's Hospital recorded approximately 24,000 emergency department visits and 3,000 hospital admissions for respiratory diseases (excluding COVID-19) among children residing in the Metropolitan Area of São Paulo. From this source population, the present study focused on two predefined diagnostic groups: unspecified acute respiratory infections, selected because they represented the most frequently recorded respiratory diagnosis in the study population, and bronchiolitis, selected because of its high clinical relevance as one of the leading causes of hospitalization among infants and young children.

### Geocoding of residential addresses

Residential addresses obtained from hospital records were evaluated for completeness and geocoded to generate geographic coordinates (latitude and longitude). Geocoding was performed in Python using the OpenCage Data API and OpenStreetMap-based geocoding procedures. The resulting coordinates were subsequently used to link patients’ residences to environmental exposure estimates derived from air pollution and meteorological monitoring data.

Geocoding quality was assessed according to the positional information provided by each geocoding source. For coordinates obtained using OpenCage, confidence scores between 8 and 10 were retained, corresponding to estimated positional accuracies ranging from <1 km to <0.25 km. Additional addresses were geocoded using OpenStreetMap at the street or POI/house-number level.

Residential addresses were evaluated for completeness, geocoded, and restricted to the Metropolitan Region of São Paulo (MRSP). Among 2,859 bronchiolitis records initially evaluated, 1,584 (55.4%) were successfully geocoded within the MRSP, 73 (2.6%) were located outside the study area, and 1,202 (42.0%) had incomplete address information. Among 13,077 unspecified acute respiratory infection records, 7,376 (56.4%) were successfully geocoded within the MRSP, 359 (2.7%) were located outside the study area, and 5,342 (40.9%) had incomplete address information.

Geocoding was performed using OpenCage and OpenStreetMap. Among the 1,584 geocoded bronchiolitis encounters, 1,328 (83.8%) were geocoded using OpenCage, including 7 with estimated accuracy <1 km, 989 < 0.5 km, and 332 < 0.25 km; the remaining 256 (16.2%) were geocoded using OpenStreetMap at street or POI/house-number level. Among the 7,376 geocoded unspecified acute respiratory infection encounters, 6,068 (82.3%) were geocoded using OpenCage, including 43 with estimated accuracy <1 km, 4,364 < 0.5 km, and 1,661 < 0.25 km; the remaining 1,308 (17.7%) were geocoded using OpenStreetMap at street or POI/house-number level.

### Environmental and urban data

Daily environmental data included PM₁₀ concentrations, O₃, temperature, and relative humidity obtained from monitoring stations operated by the São Paulo State Environmental Agency (CETESB). Seasonal indicators (autumn, winter, and spring, with summer as the reference category) were derived from the date of attendance to account for seasonal variability (12).

The environmental variables included in this study were selected because they represent short-term atmospheric exposures with well-established associations with pediatric respiratory morbidity in the scientific literature. PM₁₀, ozone, temperature, and relative humidity are routinely monitored by the São Paulo State Environmental Agency (CETESB), providing standardized daily measurements with sufficient spatial and temporal resolution for individual exposure assessment. Other environmental characteristics, such as vegetation cover, urban green spaces, land-use patterns, and built-environment indicators, may also influence respiratory health; however, these variables primarily reflect long-term contextual exposures and were beyond the scope of the present analysis, which focused on short-term environmental and climatic conditions.

### Exposure assessment and spatial interpolation

Residential addresses were geocoded to obtain latitude and longitude coordinates. Daily measurements of PM₁₀ (µg/m^3^), O₃ (µg/m^3^), temperature ( °C), and relative humidity (%) were obtained from CETESB monitoring network, which included up to 30 monitoring stations distributed across the Metropolitan Region of São Paulo, depending on the environmental variable and data availability. Days with missing environmental measurements were excluded from the exposure assessment, and no imputation procedures were performed. Environmental exposure at each residential location was estimated using the Inverse Distance Weighting (IDW) interpolation method, whereby observations from nearby monitoring stations received greater weights than those from more distant stations. The interpolation was implemented using an inverse-square distance weighting function (power = 3), considering all monitoring stations with valid observations available for the corresponding day. Continuous spatial surfaces were generated for each environmental variable and day of the study period. Individual exposure values were then assigned according to the geocoded residential address of each patient (Figure 1). Exposure corresponded to the daily mean environmental conditions recorded on the day of hospital attendance (lag 0); delayed (lagged) or cumulative exposure windows were not evaluated.

**Figure 1:**
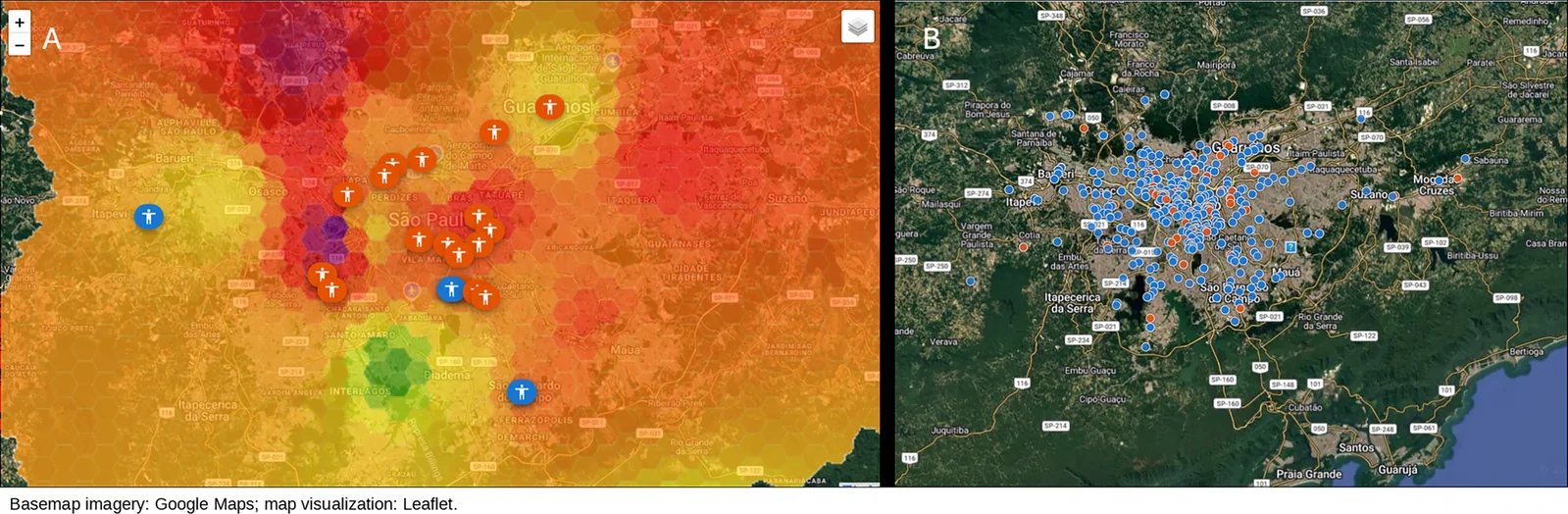
Spatial linkage between patients’ residences and environmental exposure data in the metropolitan area of São Paulo, Brazil. **(A)** Air pollution levels (O_3_) are shown for a representative day (18 February 2022) to illustrate the exposure assignment process used in the study. Red markers represent unspecified acute respiratory infections, and blue markers represent bronchiolitis cases. **(B)** Spatial distribution of all hospitalization cases included in the study for the analyzed ICD-10 respiratory diagnoses across the Metropolitan Area of São Paulo. Red markers represent unspecified acute respiratory infections, and blue markers represent bronchiolitis cases.

### Statistical analysis

Multivariable logistic regression models were constructed to evaluate the association between environmental and climatic factors and the probability that a respiratory encounter corresponded to hospitalization for a specific respiratory diagnosis. Separate models were fitted for bronchiolitis and unspecified acute respiratory infections. In each model, the outcome was coded as 1 when the respiratory encounter corresponded to hospitalization for the diagnosis of interest and as 0 for all other respiratory encounters in the study database. Accordingly, the reference category included outpatient encounters for the diagnosis of interest, outpatient encounters for other respiratory diagnoses, and hospitalizations for respiratory diagnoses other than the diagnosis of interest. This analytical approach was selected because the objective was to investigate whether environmental and climatic conditions were differentially associated with respiratory encounters corresponding to hospitalization for specific diagnoses within the broader spectrum of pediatric respiratory healthcare encounters, rather than to estimate the probability of hospitalization among children already diagnosed with a specific condition. Using all other respiratory encounters as the reference category provided a common analytical framework for comparing diagnosis-specific patterns of environmental association. Independent variables included age, sex, air pollutants (PM₁₀ and O₃), meteorological factors (temperature and relative humidity), and seasonal indicators (autumn, winter, spring, with summer as the reference category). Before model fitting, pairwise correlations among all independent variables were examined using Pearson's correlation coefficient, and multicollinearity was assessed using the Variance Inflation Factor (VIF). Odds ratios (ORs) with 95% confidence intervals (95% CI) and corresponding *p*-values were reported. All analyses were performed using SPSS (IBM SPSS Statistics, version 25), with a significance threshold of *p* < 0.05.

## Results

We analyzed the occurrence of the main ICD-10 respiratory diagnoses recorded in 2022 at Sabará Children's Hospital, a pediatric referral center in São Paulo, Brazil. Unspecified acute respiratory infections accounted for the largest number of respiratory encounters (7,376 cases) and were therefore selected as the most prevalent respiratory diagnosis in the study population. Bronchiolitis (1,584 cases), although less frequent, was included because of its high clinical relevance as one of the leading causes of hospitalization among infants and young children. Together, these diagnoses allowed the comparison of environmental associations between a highly prevalent respiratory condition and a clinically severe lower respiratory disease.

Bronchiolitis accounted for 819 outpatient visits and 765 hospitalizations, corresponding to a hospitalization proportion of 48.3%. In contrast, unspecified acute respiratory infections accounted for 7,316 outpatient visits and 60 hospitalizations, corresponding to a hospitalization proportion of 0.8%, indicating a substantially greater hospitalization burden among bronchiolitis cases.

Across the study period, unspecified acute respiratory infections were the most frequent outcome and a small fraction of hospitalizations. Most of these cases occurred in children aged 1–4 years (around half of the total), followed by those under 1 year, with a slight predominance of boys (53%). Bronchiolitis was concentrated almost entirely in infants, both outpatient visits and hospitalizations occurred predominantly in children under 1 year of age, close to two thirds of cases, and male children (59%) were more frequently affected.

The temporal analysis (Figure 2) revealed marked variations in the occurrence of respiratory diseases throughout the study period. The monthly occurrence plot demonstrated that bronchiolitis was highly concentrated during the winter and autumn months, with pronounced peaks in both outpatient visits and hospitalizations, underscoring its seasonal pattern and greater severity during colder periods. Unspecified acute respiratory infections were mostly observed in outpatient visits, with cases distributed across all months, but with a clear increase during winter. The seasonal analysis reinforced these findings, bronchiolitis was largely concentrated in winter, and unspecified acute infections reached their highest frequencies in autumn and winter, highlighting the influence of seasonal factors on pediatric healthcare demand.

**Figure 2:**
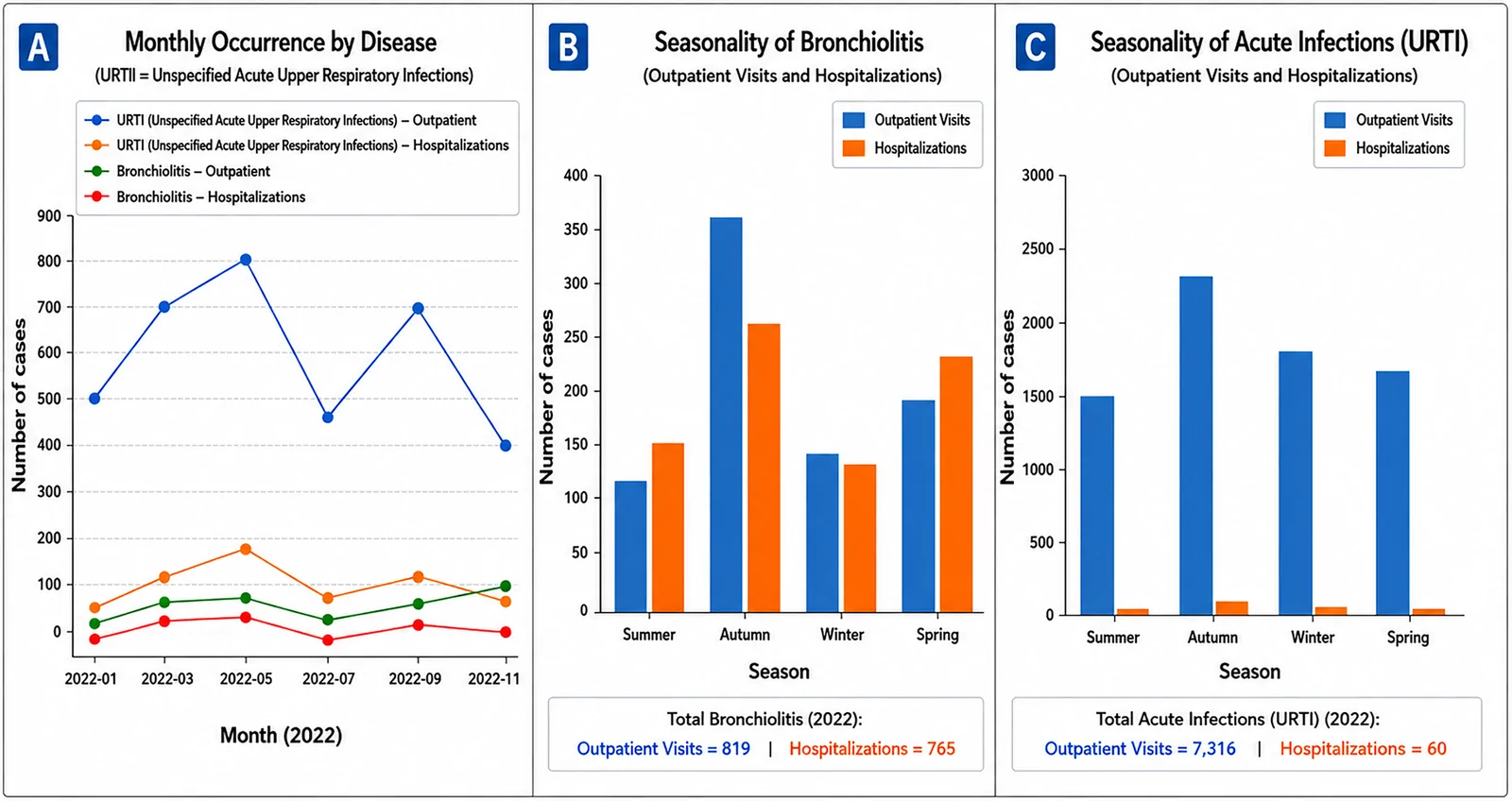
Monthly and seasonal distribution of outpatient visits and hospitalizations for pediatric respiratory diseases in the metropolitan area of São Paulo, Brazil, 2022. **(A)** Monthly occurrence of unspecified acute respiratory infections and bronchiolitis. **(B)** Seasonal distribution of bronchiolitis. (**C**) Seasonal distribution of unspecified acute respiratory infections.

Pairwise correlations among the predictor variables were generally weak to moderate, with only one pair of variables (PM₁₀ and relative humidity; r = −0.611) exceeding the predefined threshold of |r| = 0.60. All VIF values were below 5 (range: 1.60–3.24), indicating no evidence of problematic multicollinearity among the variables included in the final models.

In the logistic regression analysis (Table 1) of unspecified acute respiratory infections, none of the environmental variables reached conventional levels of statistical significance, although PM₁₀ (*p* = 0.068), O₃ (*p* = 0.056), and relative humidity (*p* = 0.069) showed positive associations with the odds that a respiratory encounter corresponded to hospitalization for unspecified acute respiratory infection, suggesting potential trends. Temperature was inversely associated with this outcome (OR = 0.887; 95% CI 0.793–0.993), indicating greater odds that a respiratory encounter corresponded to hospitalization for this diagnosis at lower temperatures. Regarding seasonality, only spring was significantly associated with greater odds of this outcome (OR = 3.386; 95% CI 1.505–7.620), while autumn and winter showed no statistically significant associations.

**Table 1:** Multivariable logistic regression models evaluating associations between environmental and seasonal factors and the odds that a respiratory encounter corresponded to hospitalization for the diagnosis of interest.

Variable	Bronchiolitis OR (95% CI)	*p*-value	URTI OR (95% CI)	*p*-value
PM_10_ (per 1 µg/m^3^)	1.007 (0.999–1.015)	0.093	1.028 (0.998–1.059)	0.068
O₃ (per 1 µg/m^3^)	**1.012** **(1.005–1.019)**	**0** **.** **001**	1.023 (0.999–1.047)	0.056
Temperature (per 1 °C)	0.982 (0.953–1.013)	0.246	**0.887** **(0.793–0.993)**	**0** **.** **036**
Relative Humidity (per 1%)	**1.016** **(1.005–1.027)**	**0** **.** **005**	1.034 (0.997–1.071)	0.069
Autumn	1.027 (0.795–1.327)	0.836	0.796 (0.292–2.170)	0.656
Winter	0.766 (0.550–1.066)	0.113	2.042 (0.615–6.774)	0.243
Spring	**1.459** **(1.145–1.859)**	**0** **.** **002**	**3.386** **(1.505–7.620)**	**0** **.** **003**

URTI – Unspecified acute respiratory infections; Odds ratios (ORs) for PM_10_, O_3_, NO, and NO2 represent a 1 μg/m^3^ increase. ORs for temperature represent a 1 °C increase, and ORs for relative humidity represent a 1% increase.

Bold values indicate statistically significant associations (*p* < 0.05).

For bronchiolitis (Table 1), higher O₃ levels (OR = 1.012; 95% CI 1.005–1.019) and relative humidity (OR = 1.016; 95% CI 1.005–1.027) were significantly associated with greater odds that a respiratory encounter corresponded to hospitalization for bronchiolitis. Temperature and PM_10_ were not significantly associated with the outcome. In terms of seasonality, spring was associated with greater odds of this outcome (OR = 1.459; 95% CI 1.145–1.859), while autumn and winter did not show statistically significant association.

Figure 3 summarizes the direction and magnitude of statistically significant associations identified in the multivariable models. Greater odds that a respiratory encounter corresponded to hospitalization for bronchiolitis were observed with higher levels of O_3_ and relative humidity, as well as during spring. In contrast, lower temperatures and spring were associated with greater odds that a respiratory encounter corresponded to hospitalization for unspecified acute respiratory infection. Figure 3 shows a visual representation highlighting the distinct patterns of association between environmental factors and specific respiratory outcomes in the pediatric population.

**Figure 3:**
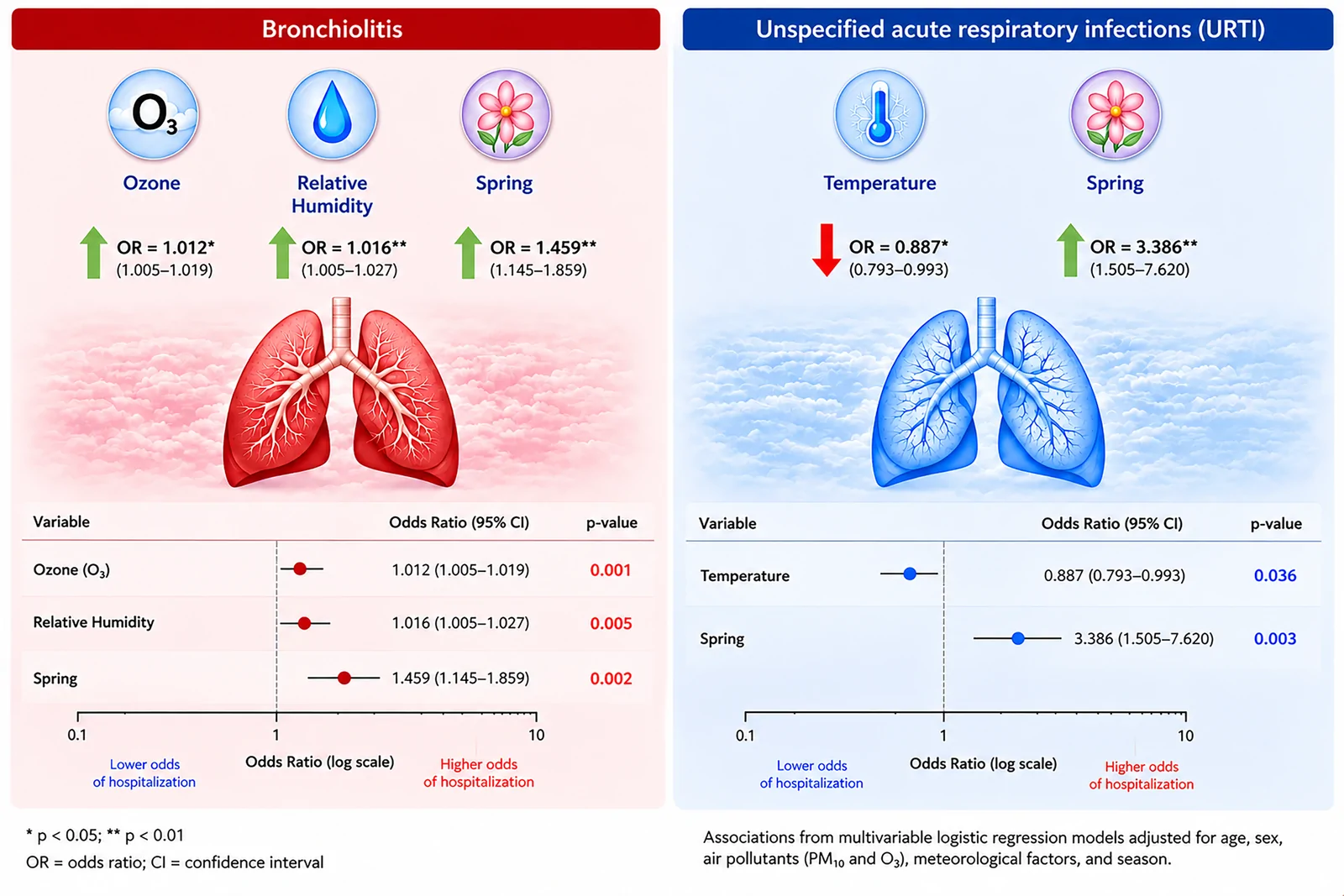
Adjusted odds ratios and 95% confidence intervals for statistically significant associations identified in the multivariable logistic regression models. Models were adjusted for age, sex, PM₁₀, O₃, temperature, relative humidity, and season. Summer was used as the reference category for seasonal variables. Continuous exposure increments were defined as 1 µg/m^3^ for PM₁₀ and O₃, 1 °C for temperature, and 1% for relative humidity.

## Discussion

In this study, we evaluated the associations of environmental and seasonal factors on hospitalizations due to respiratory diseases in the Metropolitan Region of São Paulo, Brazil. We found that unspecified acute respiratory infections were not significantly associated with air pollutants or relative humidity in hospitalization models, although borderline positive associations were observed for PM_10_, O_3_, and relative humidity. In contrast, lower temperatures were significantly associated with greater odds that a respiratory encounter corresponded to hospitalization for unspecified acute respiratory infection, while spring was also associated with greater odds of this outcome. For bronchiolitis, higher O_3_ levels, relative humidity, and spring were positively associated with the odds that a respiratory encounter corresponded to hospitalization for bronchiolitis. These findings indicate that environmental and seasonal factors may differ according to the outcome and reinforce the complexity of the interaction between climatic factors and respiratory morbidities in childhood.

Air pollution exposure has been investigated as a risk factor for hospitalization due to acute viral bronchiolitis. A recent systematic review and meta-analysis of 23 studies evaluated short, medium, and long-term exposure to PM₂.₅, PM_10_, and NO_2_ in children under 2 years of age and found the most consistent evidence for short-term exposure, with increases of 2% to 9% in the risk of hospitalization associated with these pollutants (13).

Ongoing climate change has been associated with increasing variability and disruption of traditional seasonal patterns, particularly in urban environments. As a result, the use of fixed seasonal categories may oversimplify environmental dynamics and limit the interpretation of epidemiological findings (14). In the present study, the inclusion of continuous climatic variables, such as temperature and relative humidity, allowed for a more nuanced assessment of environmental associations, potentially improving the accuracy of observed associations.

Urban living conditions are particularly evident in terms of thermal levels and air quality (e.g., PM_10_ and O_3_), as the effects of phenomena such as the urban heat island have intensified and the frequency of extreme temperature events has increased, in addition to the excessive emission of pollutants into the atmosphere (5).

Pollution, climate and seasonality conditions can have multifaceted association on health, with populations potentially being at increased or decreased risk due to the interaction of multiple factors. Identifying populations at elevated or reduced risk effects requires defining the attributes that may render them susceptible (4).

Exposure to pollutants such as PM and O_3_ has been associated with increases in hospital admissions due to respiratory diseases. These associations have been documented in short-term studies, which examine daily variations in air pollutants and health outcomes, as well as in long-term studies, which followed cohorts of individuals exposed over time (2). Evidence from controlled human exposure and epidemiological studies suggests that O₃ exposure is associated with increased respiratory symptoms and reduced lung function, particularly among individuals carrying specific genetic variants (4).

Previous studies have demonstrated short-term associations between air pollution and pediatric respiratory infections, particularly for upper respiratory tract outcomes. A case-crossover study conducted in Brisbane reported increased risks of upper respiratory infections associated with O_3_, NO_2_, and PM_₂.₅_ exposure within hours of exposure, with stronger associations observed during colder periods and nighttime. These findings are partially consistent with our results, in which O₃ was significantly associated with greater odds that a respiratory encounter corresponded to hospitalization for bronchiolitis (OR = 1.012; 95% CI 1.005–1.019) and showed a borderline association with upper respiratory infections, suggesting a relevant role of photochemical pollutants in pediatric respiratory outcomes (11).

Regarding relative humidity, previous evidence suggests a complex and non-linear relationship with respiratory morbidity. Moraes et al. (5) reported higher risks at elevated humidity levels, particularly among female children, while lower humidity was generally associated with reduced risk. In our study, relative humidity was significantly associated with greater odds that a respiratory encounter corresponded to hospitalization for bronchiolitis (OR = 1.016; 95% CI 1.005–1.027) and showed a positive, although non-significant, association with upper respiratory infections. These findings are consistent with the hypothesis that more humid conditions may be associated with a greater burden of respiratory disease, although these associations may vary according to the outcome and disease severity.

Meteorological factors, particularly temperature, have also been consistently associated with bronchiolitis dynamics. Previous studies have shown strong correlations between hospitalization rates and temperature-related variables. In our analysis, lower temperature was significantly associated with greater odds that a respiratory encounter corresponded to hospitalization for unspecified acute respiratory infection (OR = 0.887; 95% CI 0.793–0.993), consistent with previous reports describing associations between colder conditions and pediatric respiratory hospitalizations (15). Additionally, the significant association observed during spring for both bronchiolitis and upper respiratory infections suggests that seasonal transitions may be associated with changes in pediatric respiratory hospitalization patterns.

In Porto Alegre, southern Brazil, the highest number of hospital admissions for acute bronchiolitis occurs in late autumn and during winter. This pattern is consistent with the well-established seasonal distribution of bronchiolitis in Brazil, where cases typically peak during colder months, suggesting that the factors influencing these diseases are closely related to seasonal climatic variations (16).

This interpretation is further supported by our analysis of daily temperature time series. During the transition from winter to spring, particularly in September, temperatures remained relatively low, with minimum values reaching approximately 11.7 °C and a mean around 17.4 °C, indicating residual cold conditions from winter. Additionally, marked day-to-day variability was observed during this period, reflecting a transitional climatic pattern. These findings suggest that the association observed with spring may be driven by early-season conditions characterized by persistent low temperatures and thermal instability, rather than by typical springtime warmth.

Dondi et al. (7) suggested that infant bronchiolitis may be one of several adverse effects of ambient air pollution on human health, and that elevated levels of air pollutants such as PM_2.5_, C_6_H_6_, NO_2_, and PM_10_ could increase the risk of hospitalization among affected patients, thereby implying a higher likelihood of severe cases. In contrast, our results did not demonstrate significant associations with air pollution variables; rather, the observed associations were related to climate and seasonality, with certain seasons showing stronger associations. Nevertheless, policies aimed at reducing air pollution may contribute to lowering the overall burden of this disease in early childhood (7), particularly in large urban centers such as the Metropolitan Area of São Paulo.

Factors such as temperature, atmospheric pressure, vapor pressure, and precipitation were found to influence the explanatory power for most respiratory viruses in the regions analyzed in China (6). Relative humidity was a dominant factor only in the northern region but did not show significant explanatory power for most viruses in the south. Variables such as hours of sunlight demonstrated significant explanatory power for respiratory syncytial virus and influenza virus in the north, and for most other viruses in the south. Interactions between any paired factors resulted in greater explanatory power, whether bivariate or nonlinear (6). Findings such as these increasingly highlight the diverse associations between respiratory diseases and environmental factors across different parts of the world. Consistent with our study, it becomes evident that each respiratory disease may exhibit specific associations with particular climatic and pollution conditions.

Our findings are consistent with previous pediatric studies conducted in São Paulo, reinforcing the evidence that short-term environmental exposures are associated with respiratory morbidity in children. A study using distributed lag non-linear models demonstrated that temperature, relative humidity, precipitation, and PM₁₀ were significantly associated with increased hospitalizations due to respiratory diseases in children over a ten-year period, highlighting the importance of considering delayed environmental association when assessing respiratory outcomes (5). Similarly, short-term increases in ambient air pollutants, particularly O₃, NO₂, and PM₁₀, have been associated with higher pediatric hospital admissions for respiratory diseases in São Paulo, with stronger associations observed among infants (17). Another study further demonstrated that increases in NO₂ concentrations were associated with higher numbers of pediatric emergency department visits and hospital admissions for pneumonia, asthma, and bronchiolitis (18). Together, these studies support the relevance of evaluating short-term environmental exposures and reinforce the consistency of the associations observed in the present study within the context of pediatric respiratory health in large urban settings (5, 17, 18,19).

These findings have important implications for public health, particularly in large urban centers such as the Metropolitan Area of São Paulo, where exposure to air pollution and climatic variability is high. The identification of disease-specific associations suggests that prevention strategies and healthcare planning should consider the differential associations of environmental factors on respiratory conditions. Targeted interventions, including air quality control policies and seasonal preparedness measures, may help reduce the burden of severe respiratory diseases such as bronchiolitis in early childhood.

Although these findings highlight the potential contribution of environmental exposures to pediatric respiratory hospitalizations, they should be interpreted considering the characteristics of the study population. Because this study was conducted at a single private tertiary pediatric hospital, the observed associations may not be fully generalizable to all children in the Metropolitan Region of São Paulo, particularly those receiving care exclusively through the public healthcare system. Future multicenter studies including more socioeconomically diverse populations are warranted to confirm these findings.

## Conclusion

This study suggests that environmental and climatic factors are differentially associated with respiratory diseases. Specifically, higher O_3_ levels, relative humidity, and seasonal variation were associated with greater odds that a respiratory encounter corresponded to hospitalization for bronchiolitis, whereas lower temperatures and seasonal factors were associated with the corresponding outcome for unspecified acute respiratory infections. These findings highlight the importance of disaggregating respiratory outcomes, as aggregated analyses may obscure disease-specific patterns of environmental association. Incorporating disease-specific approaches into environmental health research and pediatric healthcare planning may improve prevention strategies and resource allocation in large urban centers.

## Limitations of study

This study has limitations that should be considered when interpreting the findings. First, this was a single-center study based on data from a private tertiary pediatric hospital. Although the hospital receives patients from multiple municipalities across the Metropolitan Area of São Paulo, the study population may not fully represent children receiving care in public hospitals or in other healthcare settings. Consequently, caution is warranted when extrapolating these findings to populations with different sociodemographic characteristics, healthcare access, or environmental exposure profiles. Nevertheless, the broad geographic distribution of patients allowed the evaluation of substantial spatial variability in environmental exposures across the metropolitan region. Additionally, 42.0% of bronchiolitis records and 40.9% of unspecified acute respiratory infection records could not be geocoded because of incomplete residential address information. Although this does not inherently invalidate the spatial analysis, the exclusion of these records may have introduced selection bias if address completeness was systematically associated with demographic, clinical, socioeconomic, or spatial characteristics. Second, environmental and climatic data were derived from fixed monitoring stations and interpolated for patient residences, which may not fully capture individual-level exposure, particularly for indoor environments. Consequently, some degree of exposure misclassification may have occurred, particularly because individual daily mobility, time spent indoors, and microenvironmental exposures could not be captured. Third, we did not account for potential confounders such as socioeconomic status, vaccination history, or previous comorbidities, which could influence the risk of respiratory diseases. These factors may affect children's baseline susceptibility to respiratory infections, healthcare-seeking behavior, vaccination uptake, residential location, housing conditions, and environmental exposure patterns. Consequently, residual confounding cannot be excluded, and the observed associations should be interpreted with appropriate caution. In addition, the absence of virological data, such as information on circulating respiratory viruses, limited the ability to disentangle the contribution of environmental factors from underlying viral dynamics. Because seasonal viral circulation is a major determinant of pediatric respiratory morbidity and frequently overlaps with climatic variation, part of the observed seasonal associations may reflect concurrent viral epidemics rather than environmental exposures alone. Moreover, because the study was conducted at a single private tertiary pediatric hospital, the study population may not fully represent the socioeconomic diversity of children in the Metropolitan Region of São Paulo. Therefore, caution is warranted when generalizing these findings to other healthcare settings, particularly the public healthcare system. Furthermore, studies evaluating environmental and climatic influences on respiratory diseases often identify modest association sizes because these outcomes are shaped by complex and interacting determinants. Therefore, statistically significant associations may be difficult to detect consistently, particularly in large urban settings with heterogeneous environmental exposures. In addition, the analyses were restricted to a single calendar year. Consequently, interannual variations in climatic conditions, air pollution levels, respiratory virus circulation, and healthcare demand were not evaluated, and the observed associations should be interpreted within the context of the 2022 study period. Future studies including multiple years would be valuable to assess the temporal stability of these associations. Contextual urban environmental characteristics, such as vegetation cover, green spaces, land-use patterns, and other built-environment indicators, were not included in the present models. Future studies integrating both short-term atmospheric exposures and broader urban environmental characteristics may provide a more comprehensive understanding of pediatric respiratory health. Finally, despite analyzing major respiratory diagnoses separately, some residual heterogeneity within ICD-10 categories may remain.

## Data Availability

The datasets generated and analyzed during the current study are not publicly available due to patient confidentiality, privacy considerations, and institutional restrictions imposed by Sabará Children’s Hospital. The data contain sensitive health information derived from pediatric electronic medical records. De-identified data may be made available from the corresponding author upon reasonable request and subject to approval by the hospital and the relevant ethics committee. Requests to access the datasets should be directed to William Cabral-Miranda, william.miranda@pensi.org.br.
